# Mixed-polarity random-dot stereograms alter GABA and Glx concentration in the early visual cortex

**DOI:** 10.1152/jn.00208.2019

**Published:** 2019-07-10

**Authors:** Reuben Rideaux, Nuno R. Goncalves, Andrew E. Welchman

**Affiliations:** Department of Psychology, University of Cambridge, Cambridge, United Kingdom

**Keywords:** GABA, Glx, magnetic resonance spectroscopy, mixed-polarity benefit, stereopsis

## Abstract

The offset between images projected onto the left and right retina (binocular disparity) provides a powerful cue to the three-dimensional structure of the environment. It was previously shown that depth judgements are better when images comprise both light and dark features, rather than only light or only dark elements. Since Harris and Parker (*Nature* 374: 808–811, 1995) discovered the “mixed-polarity benefit,” there has been limited evidence supporting their hypothesis that the benefit is due to separate bright and dark channels. Goncalves and Welchman (*Curr Biol* 27: 1403–1412, 2017) observed that single- and mixed-polarity stereograms evoke different levels of positive and negative activity in a deep neural network trained on natural images to make depth judgements, which also showed the mixed-polarity benefit. Motivated by this discovery, we seek to test the potential for changes in the balance of excitation and inhibition that are produced by viewing these stimuli. In particular, we use magnetic resonance spectroscopy to measure Glx and GABA concentrations in the early visual cortex of adult humans during viewing of single- and mixed-polarity random-dot stereograms (RDS). We find that participants’ Glx concentration is significantly higher, whereas GABA concentration is significantly lower, when mixed-polarity RDS are viewed than when single-polarity RDS are viewed. These results indicate that excitation and inhibition facilitate processing of single- and mixed-polarity stereograms in the early visual cortex to different extents, consistent with recent theoretical work (Goncalves NR, Welchman AE. *Curr Biol* 27: 1403–1412, 2017).

**NEW & NOTEWORTHY** Depth judgements are better when images comprise both light and dark features, rather than only light or only dark elements. Using magnetic resonance spectroscopy, we show that adult human participants’ Glx concentration is significantly higher whereas GABA concentration is significantly lower in the early visual cortex when participants view mixed-polarity random-dot stereograms (RDS) compared with single-polarity RDS. These results indicate that excitation and inhibition facilitate processing of single- and mixed-polarity stereograms in the early visual cortex to different extents.

## INTRODUCTION

Binocular stereopsis is one of the primary cues for three-dimensional (3-D) vision. It remains an important challenge to understand how the brain combines a pair of 2-D retinal images to support 3-D perception. A clue to understanding the neural computation of binocular stereopsis may be found in the observation that depth judgements are more accurate when binocular images are composed of both light and dark features, rather than just one or the other (Harris and Parker 1995).

This “mixed-polarity benefit” was originally explained on the basis that bright and dark features are processed by separate ON and OFF channels (Harris and Parker 1995). Such neural infrastructure would reduce the number of potential binocular matches in a mixed-polarity stimulus, i.e., a random-dot stereogram (RDS), by as much as half, simplifying the stereoscopic correspondence problem considerably. Separate of ON and OFF channels first appear at the bipolar cell level as ON and OFF ganglia (Nelson et al. 1978) and are maintained at the retinal ganglion and lateral geniculate nucleus level as ON and OFF center cells. However, the convergence of ON and OFF channels in V1 to form simple cells (Schiller 1992) seems to contradict this as a potential explanation for the mixed-polarity benefit.

Recently, Goncalves and Welchman (2017) showed that it is possible to capture the mixed-polarity benefit using a simple linear-nonlinear processing architecture that does not depend on separate ON and OFF channels. Thereafter, Read and Cumming (2018) proposed that the mixed-polarity benefit could arise from subtle changes in image correlation that can occur in some circumstances. Because current models of binocular processing are based on cross-correlation between the left and right eyes (Ohzawa et al. 1990), they do not consider image features, but instead compute the interocular cross-correlation between left and right images. Within this framework, higher image correlation might be expected to drive binocular cells in the primary visual cortex more strongly; thus subtle changes in interocular cross-correlation provide an explanation for the benefit that is consistent with this model. Although these explanations appear to capture the improved behavioral performance associated with mixed-polarity images, empirical evidence is needed to establish the neural basis of the effect.

Magnetic resonance spectroscopy (MRS) work with humans has provided evidence for the relationship between visual stimulation and metabolic activity in the visual cortex. For instance, the concentration of primary inhibitory and excitatory neurotransmitters, γ-aminobutyric acid (GABA) and glutamate, in the early visual cortex can be altered by viewing 2-D visual stimuli such as a flickering checkerboard (Bednařík et al. 2015; Mekle et al. 2017) or by closing one (Lunghi et al. 2015) or both eyes (Kurcyus et al. 2018). A new theoretical explanation of the mixed-polarity benefit proposes excitatory and inhibitory neural mechanisms are engaged to different extents to process single- and mixed-polarity stereoscopic images (Goncalves and Welchman 2017). However, the metabolic activity evoked by viewing these stimuli remains unknown.

In the present study we seek to test the potential for changes in the balance of excitation and inhibition that are produced by viewing single- and mixed-polarity stimuli. This follows directly from observing that single- and mixed-polarity RDS evoke different levels of positive and negative activity in a deep neural network, which also showed the mixed-polarity benefit (Goncalves and Welchman 2017). In particular, we use MRS to measure GABA concentration in the early visual cortex of human participants while they view single- and mixed-polarity RDS. We find that viewing single- and mixed-polarity RDS produces differences in the concentration of GABA. Furthermore, we find that that viewing single- and mixed-polarity RDS also produces differences in Glx, i.e., a complex comprising primary excitatory neurotransmitters glutamate (Glu) and glutamine (Gln).

## METHODS

### 

#### Participants.

Twenty healthy participants (12 women) from the University of Cambridge with normal or corrected-to-normal vision participated in the MRS experiment. The mean age was 25.5 yr (range = 19.4–40.5 yr). Participants were screened for stereoacuity using a discrimination task in which they judged the (near/far) depth profile of a RDS depicting an annulus surrounding a disk. The difference in depth between the annulus and disk was controlled using a 2-down 1-up staircase procedure, and participants were admitted into the experiment if they achieved a threshold of <1° arcmin. Participants were also screened for contraindications to MRI before the experiment. All experiments were conducted in accordance with the ethical guidelines of the Declaration of Helsinki and were approved by the University of Cambridge STEM, and all participants provided written informed consent.

#### Apparatus and stimuli.

Stimuli were programmed and presented in MATLAB (The MathWorks, Natick, MA) with Psychophysics Toolbox extensions (Brainard 1997; Pelli 1997). Stereoscopic presentation in the scanner was achieved using a “PROPixx” DLP LED projector (VPixx Technologies) with a refresh rate of 120 Hz and resolution of 1,920 × 1,080, operating in RB3D mode. The left and right images were separated by a fast-switching circular polarization modulator in front of the projector lens (DepthQ; Lightspeed Design). The onset of each orthogonal polarization was synchronized with the video refresh, enabling interleaved rates of 60 Hz for each eye’s image. MR-safe circular polarization filter glasses were worn by participants in the scanner to dissociate the left and right eye’s view of the image. Stimuli were back-projected onto a polarization-preserving screen (model 150; Stewart FilmScreen) inside the bore of the magnet and viewed via a front-surfaced mirror attached to the head coil and angled at 45° above the participants’ head. This resulted in a viewing distance of 82 cm, from which all stimuli were visible within the binocular field of view.

**Fig. 1.**
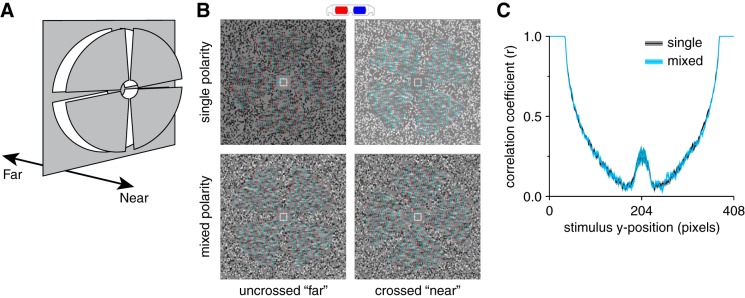
Stimuli used in the experiment. *A*: diagram of the depth arrangement of the stimuli; 4 disparity-defined wedges were simultaneously presented at either ±6 arcmin. *B*: examples of the near and far depth stimuli used in the single- and mixed-polarity conditions, designed for red-cyan anaglyph viewing. *C*: average left- and right-eye image cross-correlation as a function of stimulus *y*-axis position for single- and mixed-polarity stereogram (RDS) stimuli across a run (96 pairs of images). A paired *t*-test comparison between the cross-correlation across all positions was nonsignificant (single-polarity mean, 0.58; mixed-polarity mean, 0.58; *t*_95_ = 0.82, *P* = 0.413). Shaded regions show SE.

Stimuli consisted of RDS (12° × 12°) on a mid-gray background surrounded by a static grid of black and white squares intended to facilitate stable vergence. Dots in the stereogram followed a black or white Gaussian luminance profile, subtending 0.07° at half maximum. There were 108 dots/deg^2^, resulting in ~38% coverage of the background. Dots were allowed to overlap, and they occluded previously positioned dots where this occurred. In the center of the stereogram, four wedges were equally distributed around a circular aperture (1.2°), each subtending 10° in the radial direction and 70° in polar angle, with a 20° gap between wedges (Fig. 1*A*). Dots comprising the wedges were offset by 10 arcmin between the left and right eyes, whereas the remaining dots had zero offset. Stimuli were presented for 1.8 s and separated by 0.2-s interstimulus intervals consisting of only the background and fixation cross. To reduce adaptation, we applied a random polar rotation on each presentation to the set of wedges such that the disparity edges of the stimuli were in different locations for each stimulus presentation (i.e., a rigid body rotation of the four depth wedges together around the fixation point). Every five presentations, we reversed the sign of the disparity of the wedges (crossed and uncrossed; Fig. 1*B*). At a given time point, all wedges were presented the same disparity. In the center of the wedge field, we presented a fixation square (side length = 1°) paired with horizontal and vertical nonius lines.

Two conditions were run: single- and mixed-polarity. In the single-polarity condition, the stimulus comprised uniform polarity dots and alternated every presentation; e.g., even-numbered presentations comprised white dots and odd-numbered presentations comprised black dots. In the mixed-polarity condition, the stimulus comprised equal proportions of randomly interspersed black and white dots (Fig. 1*B*). Read and Cumming (2018) have proposed that the mixed-polarity benefit arises from differences in the interocular cross correlation between some single- and mixed-polarity RDS. The difference in correlation arises from an interaction between the range of luminance in the stereograms and the variability of the binocular disparity. To avoid this potential confound, we designed the stimuli such that the variability of the binocular disparity in the images was low; binocular disparity was either 0 or ±10 arcmin. To compare the cross-correlation of the single- and mixed-polarity stimuli, we calculated Pearson’s *r* for each (left and right eye) pair of horizontal lines of pixels, from the top to the bottom of the stimulus, for all stimuli generated in a session (96 pairs of images). Figure 1*C* shows the results of the comparison, confirming that there was no significant difference.

#### Vernier task.

During active (single/mixed-polarity condition) scans, participants performed an attentionally demanding Vernier task at fixation. This task served two purposes: *1*) it ensured consistent attentional allocation between conditions, and *2*) it provided a subjective measure of eye position, allowing us to assess whether there were any systematic differences in eye vergence between conditions. Participants were instructed to fixate a central cross hair fixation marker. The fixation marker consisted of a white square outline (side length 30 arcmin) and horizontal and vertical nonius lines (length 22 arcmin). One horizontal and one vertical line were presented to each eye to promote stable vergence and to provide a reference for a Vernier task (Popple et al. 1998). The Vernier target line subtended 6.4 arcmin in height by 2.1 arcmin in width and was presented at seven evenly spaced horizontal offsets of between ±6.4 arcmin for 500 ms (with randomized onset relative to stimulus) on 33% of presentations. Participants were instructed to indicate, by button press, on which side of the central upper vertical nonius line the target appeared, and the target was presented monocularly to the right eye.

#### Procedure.

Participants underwent four MR spectroscopic acquisitions: an initial resting acquisition, followed by two active acquisitions, separated by a second resting acquisition that was half the length of the initial resting acquisition. The primary purpose of the second resting acquisition was to allow metabolite concentrations to return to a baseline state between active acquisitions. During resting acquisitions, participants were instructed to close their eyes. During active acquisitions, participants performed the Vernier task while viewing either single or mixed-polarity stereograms. The order of (single/mixed) active acquisitions was counterbalanced across participants.

#### Magnetic resonance spectroscopy.

Magnetic resonance scanning was conducted on a 3T Siemens Prisma equipped with a 32-channel head coil. Anatomical T1-weighted images were acquired for spectroscopic voxel placement with an MP-RAGE sequence. For detection of GABA, spectra were acquired using a MEGA-PRESS sequence: echo time = 68 ms, repetition time = 3,000 ms; 256 transients of 2,048 data points were acquired in 13-min experiment time; a 14.28-ms Gaussian editing pulse was applied at 1.9 (ON) and 7.5 (OFF) parts per million (ppm); water unsuppressed 16 transients. Water suppression was achieved using variable power with optimized relaxation delays (VAPOR) and outer volume suppression (OVS). Automated shimming followed by manual shimming was conducted to achieve ~12-Hz water linewidth.

**Fig. 2.**
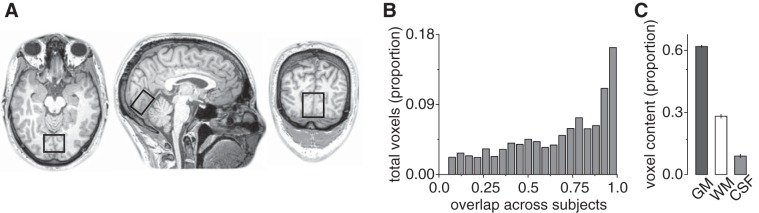
Magnetic resonance spectroscopy (MRS) voxel location across participants. *A*: a representative example of the location of the MRS voxel is shown. *B*: proportion of the total anatomical MRI voxels within the MRS voxel that are common between participants, as a function of the number of participants to which they are common; e.g., ~16% of anatomical voxels within the MRS voxel are found in the same MNI location for all participants, ~11% are found in 19/20 participants, etc. *C*: average proportion of gray matter (GM), white matter (WM), and cerebral spinal fluid (CSF) contained in the MRS voxels.

Spectra were acquired from a location targeting early visual cortex, i.e., V1/V2 (Fig. 2*A*). The voxel (3 × 3 × 2 cm) was placed medially in the occipital lobe, with the lower face aligned with the cerebellar tentorium and positioned to avoid including the sagittal sinus and to ensure it remained within the occipital lobe. To assess the consistency of voxel placement across participants, the voxel location was used to mask a 3-D anatomical image of the participants’ brain. The resulting image was then transformed into Montreal Neurological Institute (MNI) space, and the degree of overlap between voxel masks across participants was calculated (Fig. 2*B*). Furthermore, we quantified the range of gray matter (GM), white matter (WM), and cerebral spinal fluid (CSF) in each voxel (Fig. 2*C*). This was then used to apply a CSF correction (Kreis et al. 1993) to the metabolite measurements with the following equation:

(1) $${\text{C}}_{\text{tisscorr}}=\frac{{\text{C}}_{\text{meas}}}{f_{\text{GM}}+f_{\text{WM}}}$$

where C_tisscorr_ and C_meas_ are the CSF-corrected and uncorrected metabolite concentrations, respectively, and *f*_GM_ and *f*_WM_ are the proportions of GM and WM within the voxel, respectively. Segmentation was performed using the Statistical Parametric Mapping toolbox for MATLAB (SPM12; https://www.fil.ion.ucl.ac.uk/spm/). The DICOM of the voxel location was used as a mask to calculate the volume of each tissue type (GM, WM, CSF).

Spectral quantification was conducted with GANNET (Baltimore, MD), a MATLAB toolbox designed for analysis of GABA MEGA-PRESS spectra, modified to fit a separate double-Gaussian to each GABA+ (GABA and co-edited macromolecules) and Glx (a complex comprising Glu and Gln) peaks. Individual spectra were frequency- and phase-corrected to the choline and creatine peaks. Total creatine (tCr) signal intensity was determined by fitting a single mixed Gaussian-Lorentzian peak to the mean nonedited spectra, whereas water signal intensity was determined by fitting a single mixed Gaussian-Lorentzian peak to the mean of the 16 water unsuppressed transients. ON and OFF spectra were subtracted to produce the edited spectrum, from which GABA+ and Glx signal intensities were modeled off double-Gaussian peaks. Intensities of GABA+ and Glx were normalized to the commonly used internal reference tCr (Jansen et al. 2006), yielding relative concentration values (i.e., GABA+:tCr and Glx:tCr). The tCr signal is acquired within the same MEGA-PRESS acquisitions as the target metabolites; thus normalization of GABA+ and Glx to tCr minimizes the influence of subject movement during the scan and eliminates the effects of chemical shift displacement (Mullins et al. 2014). Furthermore, if changes occur during the acquisition that alter the entire spectrum (e.g., changes in signal strength, line width, or dilution associated with changes in blood flow; Ip et al. 2017) this will produce no change in the ratio of target metabolites to tCR. As an additional control, GABA+ and Glx were also normalized to water.

Table 1 shows the average full width at half maximum (FWHM) and frequency drift of the spectra across conditions. The fitting residuals for GABA+, Glx, and tCr were divided by the amplitude of their fitted peaks to produce normalized measures of uncertainty. There were no significant differences in the fitting residuals between conditions for any of these metabolites, and the average for each was relatively low (~5% for GABA+/Glx and ~3% for tCr; Table 1; Mullins et al. 2014; Rowland et al. 2016). Individual GABA+ and Glx peak model fits are shown in Supplemental Fig. S1 (https://github.com/ReubenRideaux/supplementary_material).

**Table 1.** Measures of spectral quality and fit error

Condition	FWHM			Frequency drift, std. Hz	Fit Error		
	GABA	Glx	Creatine		GABA	Glx	Creatine
Resting	27.71 ± 2.84	15.46 ± 1.94	9.78 ± 1.74	1.12 ± 0.53	5.17 ± 0.86	3.03 ± 0.73	5.84 ± 1.32
Single	27.87 ± 2.74	15.82 ± 1.34	10.28 ± 1.25	0.73 ± 0.29	4.86 ± 0.94	2.92 ± 0.75	5.57 ± 0.92
Mixed	27.65 ± 2.66	16.31 ± 1.37	10.37 ± 1.25	0.77 ± 0.42	4.85 ± 0.98	3 ± 0.86	5.54 ± 0.89

Values are means ± SD. FWHM, full width at half maximum; Glx, a complex comprising Glu and Gln; std., standard.

For the dynamic analysis, we used a sliding window (width, 128 acquisitions; step size, 1 acquisition) to measure average GABA+ and Glx concentrations as they changed while participants viewed single- and mixed-polarity stimuli (256 acquisitions/13 min). Reducing the number of acquisitions in the averaged spectra reduced the signal-to-noise ratio. Thus, before the dynamic analysis was run, metabolite concentration data were screened to remove noisy and/or spurious quantifications. In particular, we removed data points that were >4 SD from the mean at each time point. This resulted in the removal of zero data points from the GABA+ data set and 0.3% of data points from the Glx data set. To remove spurious significant differences in the time course between conditions, a cluster correction was applied. Clusters were defined by the sum of their constituent (absolute) *t* values and compared with a null hypothesis distribution of clusters produced by shuffling the condition labels (1,000 permutations). Clusters below the 95th percentile of the null hypothesis distribution were disregarded.

## RESULTS

### 

#### GABA.

**Fig. 3.**
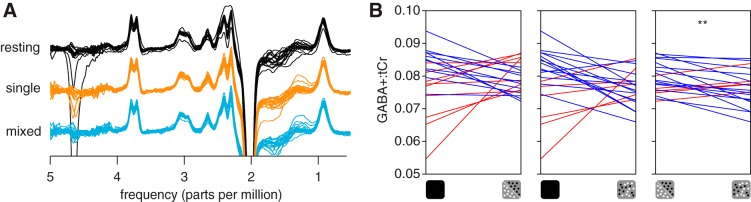
Average spectra and GABA+ (GABA and co-edited macromolecules) measurements. *A*: individual difference spectra acquired while participants were at rest or viewing single- and mixed-polarity random-dot stereograms (RDS). *B*: GABA+ concentration referenced to total creatine (GABA+:tCR) from a voxel targeting early visual cortex while observers were at rest or viewing single- or mixed-polarity RDS. Each panel shows individual concentration values paired between conditions: *left*, resting-single; *middle*, resting-mixed; *right*, single-mixed. Red and blue lines indicate increasing and decreasing concentration, respectively. **P < 0.01, significant difference.

The GABA+ peaks were clearly visible within the spectra at ~3 ppm (Fig. 3*A*). We quantified the concentration of the primary inhibitory neurotransmitter GABA and found that GABA+ concentration was significantly lower when participants viewed mixed-polarity stereograms compared with single-polarity stereograms (paired *t*-test, *t*_19_ = 2.99, *P* = 0.008; Fig. 3, *B* and *C*). A possible concern might be that the observed change in GABA+:tCr concentration relates to changes in tCr, rather than GABA+. However, we found the same result when GABA+ was referenced to water (paired *t*-test, *t*_19_ = 2.89, *P* = 0.009).

To assess the direction of metabolic change from “baseline” that viewing single- and mixed-polarity RDS produced, we acquired an initial resting measurement where participants were instructed to close their eyes. Despite the significant difference in GABA+ observed between viewing of single- or mixed-polarity stereograms, the concentrations measured in these conditions did not significantly differ from the rest measurement (Fig. 3, *B* and *C*).

**Fig. 4.**
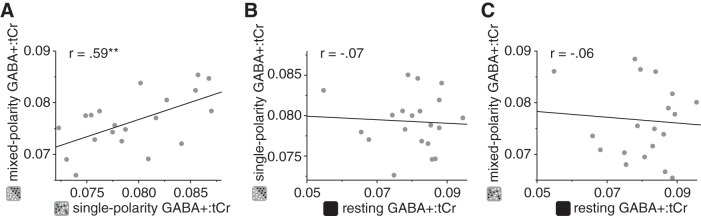
Within-subject correlations of GABA+ (GABA and co-edited macromolecules) concentration referenced to total creatine (GABA+:tCr) in the early visual cortex while participants were at rest or viewing single- or mixed-polarity random-dot stereograms. To assess within-subject consistency of metabolite concentrations, we compared GABA+ correlations between active conditions (single- and mixed-polarity; *A*) and between active conditions and rest (*B* and *C*). **P < 0.01, significant correlation score.

An explanation for this might be that there was variability in the signal during the resting acquisition that was not present during the active acquisitions (Ip et al. 2017). For example, fixation and attention were controlled in the single- and mixed-polarity conditions by requiring participants to perform a demanding Vernier task at fixation. By contrast, we instructed participants to close their eyes during the resting acquisition, but we can confirm neither the extent to which they heeded this instruction nor the focus of their attention. Previous work has shown that GABA and Glx concentrations in the primary visual cortex can be influenced by whether the eyes are open or closed (Kurcyus et al. 2018); thus this could have increased variability in the rest condition. The variance in the resting condition was numerically larger in the resting conditions than the active conditions, but not significantly. However, we found that the correlation between GABA+ concentration when participants viewed single- and mixed-polarity RDS (Pearson correlation, *r* = 0.59, *P* = 0.006; Fig. 4*A*) was significantly higher than that between resting GABA+ and active GABA+ (single: *z* = 2.17, *P* = 0.030; mixed: *z* = 2.13, *P* = 0.032), the latter of which were not significantly different from zero (single: *r* = −0.07, *P* = 0.76; mixed: *r* = −0.06, *P* = 0.80; Fig. 4, *B* and *C*). That is, individuals’ GABA+ concentration was similar between different “active” conditions, but it was not similar when active conditions were compared with rest. This could be caused by additional sources of variability in the rest condition and may explain why we did not detect a significant change in GABA+ concentration between rest and active conditions.

**Fig. 5.**
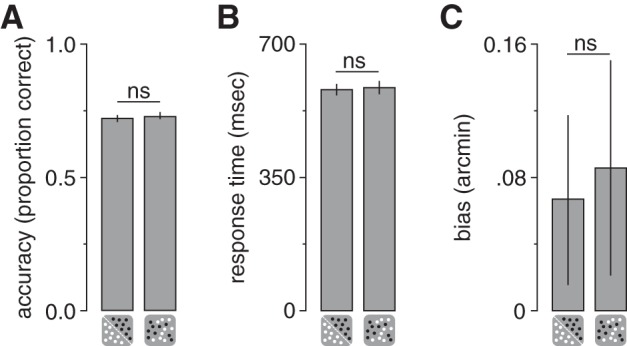
Vernier task performance during single- and mixed-polarity viewing acquisitions. *A* and *B*: comparisons of accuracy (*A*) and response time (*B*) on a Vernier task performed while participants viewed single- and mixed-polarity stimuli. *C*: bias (deviation from the desired vergence position) in participants’ judgments. Error bars indicate SE; ns, no significant difference.

A possible concern might be that the observed difference in GABA+ concentration between active conditions was due to differences in attentional allocation or eye movements when participants viewed single- and mixed-polarity stereograms. However, we found no evidence of a difference in performance on the attentionally demanding Vernier task between conditions, either in accuracy (paired *t*-test, *t*_18_ = 0.93, *P* = 0.36; Fig. 5*A*) or in response time (paired *t*-test, *t*_18_ = 0.60, *P* = 0.57; Fig. 5*B*). For each participant we fitted a cumulative Gaussian to the proportion of “right” responses as a function of the horizontal offsets of the targets, to obtain bias measurements. Bias (deviation from the desired vergence position) in participants’ judgments was not significantly different between conditions (paired *t*-test, *t*_18_ = 0.31, *P* = 0.76; Fig. 5*C*) and was not significantly different from zero (paired *t*-test, single: *t*_18_ = 1.34, *P* = 0.20; mixed: *t*_18_ = 1.36, *P* = 0.19). Performance on the Vernier task therefore suggests that participants were able to maintain stable eye vergence equally well between single- and mixed-polarity conditions (Popple et al. 1998).

**Fig. 6.**
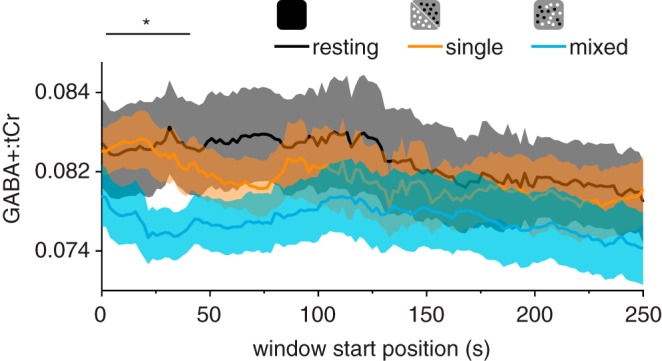
Dynamic GABA+ (GABA and co-edited macromolecules) concentration referenced to total creatine (GABA+:tCr) in early visual cortex. We used a sliding window (kernel size, 128 acquisitions; step size, 1 acquisition/2 s) to measure GABA+ concentration as it changed while participants were at rest or viewing single- or mixed-polarity stimuli (256 acquisitions/13 min); thus the first time point indicates the concentration of GABA+ measured from spectra averaged over the first 128 acquisitions/256 s. The horizontal line at the *top* of the plot indicates periods where the difference in concentration while participants were viewing single- and mixed-polarity stimuli was significant, following cluster correction. Shaded regions show SE. *P < 0.05, continuous period of significant differences.

Having established that viewing single- and mixed-polarity stereograms produced differences in GABA+ concentration in the early visual cortex, we tested whether the difference in GABA+ concentration between conditions was stable or changed during the presentation. We used a sliding window to measure GABA+:tCr as it dynamically changed over the course of the acquisition (Branzoli et al. 2013; Schaller et al. 2013). We found that the difference in concentration when participants viewed single- and mixed-polarity stereograms was greatest early in the acquisition (*P* < 0.05 from *steps 2–22*, *P*_min_ = 5.4e^−4^; Fig. 6). This suggests that visual stimulation altered GABA+ concentration most during the first half of the presentation (~7 min), after which it began to return to its initial state.

We counterbalanced the active condition order across participants to balance potential changes in metabolic concentration over the course of the scan that were unrelated to the stimulus presentation. However, given that we found evidence for change in GABA+ concentration over the course of the presentation sequence, we tested whether there was a consistent change in GABA+ concentration over the course of the presentation by separately comparing data from participants who viewed either the single- or mixed-polarity RDS first. We found a main effect of (single-/mixed-polarity) condition (*F*_1,19_ = 9.35, *P* = 0.007; Supplemental Fig. S2a; https://github.com/ReubenRideaux/supplementary_material), but no effect of condition order (*F*_1,19_ = 0.01, *P* = 0.94) and no interaction (*F*_1,19_ = 2.04, *P* = 0.17). These results suggest that GABA+ concentration did not change, independently of stimulus presentation, over the course of the two active conditions. Furthermore, we found no significant difference in GABA+ concentration measured in the first and (shorter) second rest periods (paired *t*-test, *t*_19_ = 0.14, *P* = 0.89).

#### Glx.

**Fig. 7.**
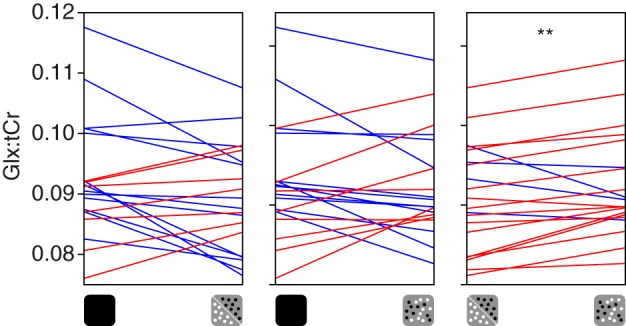
Glx (a complex comprising Glu and Gln) measurements. Glx concentration referenced to total creatine (Glx:tCr) was measured from a voxel targeting early visual cortex while observers were at rest or viewing single- or mixed-polarity random-dot stereograms. Each panel shows individual concentration values paired between conditions: *left*, resting-single; *middle*, resting-mixed; *right*, single-mixed. Red and blue lines indicate increasing and decreasing concentration, respectively. **P < 0.01, significant difference.

We found that GABA+ concentration measured from a voxel targeting V1 and V2 was different when participants viewed single- and mixed-polarity stereograms, suggesting that there was different involvement of inhibitory systems in the processing of these stimuli. We then compared the concentration of Glx, a complex comprising Glu (the primary excitatory neurotransmitter) and Gln, between these conditions. The Glx peaks were clearly visible in the spectra at ~3.8 ppm (Fig. 3*A*). We found that Glx was significantly higher in the mixed-polarity condition (paired *t*-test, *t*_19_ = 2.35, *P* = 0.029; Fig. 7, *A* and *B*). Furthermore, we found the same result when Glx was referenced to water (paired *t*-test, *t*_19_ = 2.47, *P* = 0.023).

**Fig. 8.**
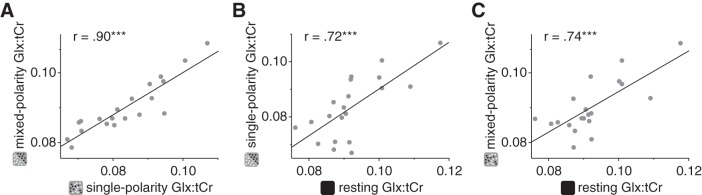
Within-subject correlations of Glx (a complex comprising Glu and Gln) concentration referenced to total creatine (Glx:tCr) in the early visual cortex while participants were at rest or viewing single- or mixed-polarity random-dot stereograms. To assess within-subject consistency of metabolite concentrations, we compared Glx correlations between active conditions (*A*) and between active conditions and rest (*B* and *C*). ***P < 0.001, significant correlation score.

**Fig. 9.**
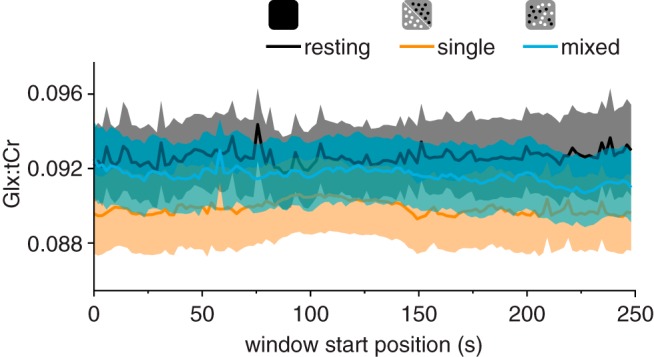
Dynamic Glx (a complex comprising Glu and Gln) concentration referenced to total creatine (Glx:tCr) in early visual cortex. We used a sliding window (kernel size, 128 acquisitions; step size, 1 acquisition) to measure GABA+ (GABA and co-edited macromolecules) concentration as it changed while participants were at rest or viewing single- or mixed-polarity stimuli (256 acquisitions/13 min). No significant differences survived cluster correction. Shaded regions are SE.

Similar to the GABA+ results, comparison of Glx concentration measured in the viewing conditions with that measured at rest yielded no significant differences. However, unlike the GABA+ results, the correlation between Glx concentration while participants viewed single- and mixed-polarity RDS (Pearson correlation, *r* = 0.90, *P* = 4.3e^−8^; Fig. 8*A*) was not significantly higher than that between resting Glx and active Glx (single: *z* = 1.72, *P* = 0.085; mixed: *z* = 1.61, *P* = 0.108; Fig. 8, *B* and *C*). This may be because Glu is only a component of the Glx complex, and the other component (Gln) may be more stable between resting and active periods. Additionally, unlike GABA, the concentration of Glx has a significant contribution from the metabolic pool, which is likely to remain relatively stable over time. For the dynamic analysis of Glx concentration, we found that the difference remained stable during the acquisition (Fig. 9). Similarity, analysis of condition order for Glx concentration revealed a main effect of (single-/mixed-polarity) condition (*F*_1,19_ = 5.88, *P* = 0.026; Supplemental Fig. S2b; https://github.com/ReubenRideaux/supplementary_material), but no effect of order (*F*_1,19_ = 1.51, *P* = 0.23) and no interaction (*F*_1,19_ = 1.23, *P* = 0.28). There was no significant difference in Glx concentration measured in the first and second rest periods (paired *t*-test, *t*_19_ = 1.77, *P* = 0.09).

**Fig. 10.**
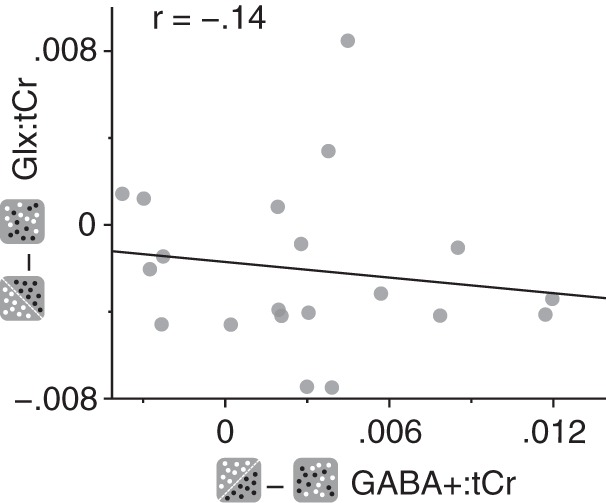
Difference in Glx (a complex comprising Glu and Gln) concentration referenced to total creatine (Glx:tCr) between single- and mixed-polarity viewing conditions as a function of the difference in GABA+ (GABA and co-edited macromolecules) concentration referenced to total creatine (GABA+:tCr) between single- and mixed-polarity viewing conditions.

We found that concentrations of GABA+ in the early visual cortex were significantly lower when participants viewed mixed-polarity stereograms compared with single-polarity stereograms. By contrast, we found the opposite effect for Glx; concentrations were higher when participants viewed mixed-polarity stereograms. It is possible that a decrease in one may have coincided with an increase in the other, e.g., to maintain a particular balance of inhibition and excitation. However, we found no evidence for a correlation between the difference in GABA+ and Glx concentrations between single- and mixed-polarity viewing conditions (Pearson correlation, *r* = −0.14, *P* = 0.56; Fig. 10).

## DISCUSSION

Depth judgements are more accurate when binocular images are composed of both light and dark features, rather than just one or the other (Harris and Parker 1995; Read et al. 2011). This finding was initially interpreted as evidence of separate ON and OFF binocular channels; however, contradictory physiological (Schiller 1992), behavioral (Read et al. 2011), and theoretical evidence (Goncalves and Welchman 2017; Read and Cumming 2018) has cast doubt on this hypothesis. In the present study we tested the potential for changes in the balance of excitation and inhibition that are produced by viewing these single- and mixed-polarity RDS. This follows directly from the recent observation that these stimuli evoke different levels of positive and negative activity in a deep neural network, which also showed the mixed-polarity benefit (Goncalves and Welchman 2017). We show that GABA+ concentration measured in the early visual cortex is lower when participants view mixed-polarity stereograms than when they view single-polarity stereograms. Furthermore, we show the opposite pattern of results for the Glx (Glu-Gln) complex.

The finding that GABA+ concentration was different when participants viewed single- and mixed-polarity stereograms indicates that these stimuli evoke different levels of suppressive activity in the early visual cortex. Given the current understanding of MRS-measured changes in GABA and its relationship to neural function, interpreting these results as unambiguous evidence for increased suppressive activity in one condition or the other is challenging. For example, increased suppressive activity demands a corresponding increase in GABA synthesis, which may expand the “pool” of GABA that can be detected by MRS. However, when GABA is released from the cell body into the synapse, it becomes bound to GABA receptors, which broadens its resonance and makes it less detectable with MRS (Jahnke et al. 2002; Xu et al. 2000). Whereas the former point predicts an increase in MRS-measured GABA concentration, the latter predicts a reduction. Establishing the directionality of the GABA+ change is further complicated because we did not detect a metabolic difference in either viewing condition from baseline.

In addition to a difference in GABA+ concentration, we also observed a difference in Glx (a complex comprising Glu and Gln) between single- and mixed-polarity stereogram viewing conditions. Given that Gln is a primary source of GABA synthesis (Patel et al. 2001; Paulsen et al. 1988; Rae et al. 2003), the change we observed in Glx may be due to differences in the activity of the inhibitory system, as evidenced by the change in GABA+ concentration. However, we found no evidence supporting this interpretation; that is, there was no relationship between the magnitude of change in these metabolites between participants. Alternatively, the difference in Glx may be attributed to altered metabolic and/or neurotransmitter synthesis of Glu due to differences in activity of the excitatory system during viewing of single-/mixed-polarity stereograms. This explanation is supported by MRS work with phantoms that estimate the signal contribution to Glx in the MEGA-PRESS difference spectrum as either equal parts Glu and Gln (van Veenendaal et al. 2018) or primarily Glu (Shungu et al. 2013). Although Glu acts as an excitatory neurotransmitter, it also plays a role in energy metabolism, and the current data cannot distinguish the relative contribution of these pools of Glu to the observed change in Glx.

These results are broadly consistent with the recent observation that single- and mixed-polarity stereograms evoke different levels of positive and negative activity from a convolutional neural network trained on natural stereo images to make depth judgements (Goncalves and Welchman 2017). Critically, this neural network also reproduced the mixed-polarity benefit, suggesting that the difference in positive/negative activity may underlie this puzzling phenomenon.

Read and Cumming (2018) have proposed that the mixed-polarity benefit arises from subtle changes in image correlation under some restricted circumstances. Given that the image correlation was the same between single- and mixed-polarity conditions, the difference in neurotransmitter concentration cannot be attributed to this hypothesis. It is also unlikely that differences in attention between conditions provide an explanation for the changes in metabolic concentrations, because we found equivalent performance for the psychophysical task that participants performed at the fixation marker. We cannot, however, rule out the possibility that the observed effects were caused by monocular differences between the stimuli. For instance, the luminance of the stimuli in the single- and mixed-polarity conditions was the same when averaged over any two consecutive presentations, i.e., all white followed by all black is equivalent to two mixed presentations, but between consecutive presentations, the mean luminance of stimuli in the single-polarity condition varied more than in the mixed-polarity condition. Because the approach we used was between conditions with repeated measurements, we were limited in the extent to which we could sample from more than one voxel location. Thus we cannot rule out the possibility that the changes in GABA+ and Glx observed between single- and mixed-polarity conditions were restricted to the early visual cortex. However, according to previous literature, V1 is the most likely location in which we would expect to observe differences in the concentration of these metabolites in response to single- and mixed-polarity RDS.

A limitation of MRS is that it measures total concentration of neurochemicals within a localized region and cannot distinguish between intracellular and extracellular pools of GABA. This is relevant, because these pools are thought to have different roles in neuronal function. In the present study we show that GABA+ concentration in the early visual cortex is different when participants view single- or mixed-polarity RDS, indicating changes in the level of intracellular vesicular GABA, which drives neurotransmission (Belelli et al. 2009). However, MRS also measures extracellular GABA, which maintains tonic cortical inhibition (Martin and Rimvall 1993) and is unlikely to be altered by our experimental manipulations. Although the magnitude of GABA+ difference we observed was consistent with previous work (Bednařík et al. 2015; Mekle et al. 2017), this may explain its meager size (4%).

Dynamic analysis of GABA+ and Glx concentrations revealed that the difference in GABA+ concentration was largest early in the scan, whereas the difference in Glx concentration appeared to be relatively stable throughout. These results are broadly consistent with previous work (Bednařík et al. 2015; Chen et al. 2017; Lin et al. 2012); however, whereas we found early differences in GABA+ concentration, i.e., in the first 7 min of stimulation, (Chen et al. 2017) found the maximum difference after 5 min of hand clenching. The distinct time courses found for GABA+ and Glx when participants viewed single- and mixed-polarity stereograms may reflect differences in the extent to which inhibitory and excitatory systems were engaged. In particular, the early difference in GABA+ concentration may suggest a large difference in evoked inhibitory activity, which was subsequently accommodated. By contrast, the small but consistent difference in Glx concentration may reflect a smaller, more stable, difference in evoked excitatory activity.

To summarize, we found differences in GABA+ and Glx concentrations when participants viewed single- and mixed-polarity RDS. These results indicate different levels of inhibitory and excitatory activity are evoked by the stereoscopic computation of these stimuli and may hold the key to understanding why depth judgements are better for stereograms comprising both light and dark features.

## GRANTS

This work was supported by the Leverhulme Trust Grant ECF-2017-573, Issac Newton Trust Grant 17.08(o), and Wellcome Trust Grant 095183/Z/10/Z.

## DISCLOSURES

No conflicts of interest, financial or otherwise, are declared by the authors.

## AUTHOR CONTRIBUTIONS

N.R.G. and A.E.W. conceived and designed research; R.R. and N.R.G. performed experiments; R.R. analyzed data; R.R., N.R.G., and A.E.W. interpreted results of experiments; R.R. prepared figures; R.R. drafted manuscript; R.R., N.R.G., and A.E.W. edited and revised manuscript; R.R., N.R.G., and A.E.W. approved final version of manuscript.
